# On-Glass Integrated SU-8 Waveguide and Amorphous Silicon Photosensor for On-Chip Detection of Biomolecules: Feasibility Study on Hemoglobin Sensing

**DOI:** 10.3390/s21020415

**Published:** 2021-01-08

**Authors:** Alessio Buzzin, Rita Asquini, Domenico Caputo, Giampiero de Cesare

**Affiliations:** Department of Information Engineering, Electronics and Telecommunications, University of Rome “La Sapienza”, Via Eudossiana 18, 00184 Rome, Italy; rita.asquini@uniroma1.it (R.A.); domenico.caputo@uniroma1.it (D.C.); giampiero.decesare@uniroma1.it (G.d.C.)

**Keywords:** biosensor, Lab-on-Chip, amorphous silicon, photodetector, SU-8, waveguide, hemoglobin

## Abstract

An optoelectronic, integrated system-on-glass for on-chip detection of biomolecules is here presented. The system’s working principle is based on the interaction, detected by a hydrogenated amorphous silicon photosensor, between a monochromatic light travelling in a SU-8 polymer optical waveguide and the biological solution under analysis. Optical simulations of the waveguide coupling to the thin-film photodiode with a specific design were carried out. A prototype was fabricated and characterized showing waveguide optical losses of about 0.6 dB/cm, a photodiode shot noise current of about 2.5 fA/$\sqrt{\mathrm{Hz}}$ and responsivity of 495 mA/W at 532 nm. An electro-optical coupling test was performed on the fabricated device to validate the system. As proof of concept, hemoglobin was studied as analyte for a demonstration scenario, involving optical simulations interpolated with experimental data. The calculated detection limit of the proposed system for hemoglobin concentration in aqueous solution is around 100 ppm, in line with colorimetric methods currently on the market. These results show the effectiveness of the proposed system in biological detection applications and encourage further developments in implementing these kinds of devices in the biomedical field.

## 1. Introduction

Over recent decades, biosensing systems have spread and evolved due to constant technology improvement [1,2]. Among all the possible ways to study and analyze biological material, mechanical [3], chemical [4] and optical [5] techniques are the most common to assess and detect an analyte in biological environment. Although conventional “extract and evaluate” detection methods provide high performance and significant specificity, their lengthy procedures are usually performed by bulky and expensive instrumentation. The need for practical, rapid and inexpensive detection propositions justifies new efforts in the development of portable, highly integrated and cost-effective devices that can perform in situ operations [6,7]. Optical investigations of the analyte features based on monitoring its optical properties (such as absorption, fluorescence, chemiluminescence or complex refractive index) can be performed by small, compact biosensors or Lab-on-Chip (LOC) devices [8,9,10], assuring quick response time and reduced reagents’ consumption without sacrificing high performance in terms of sensitivity and reliability [11,12,13]. These microdevices can recognize a change in the complex refractive index of a biological sample, and relate it to the analyte concentration in a non-destructive inspection, without hazardous chemicals, avoiding the need for large sample volumes and for their subsequent disposal. Constant efforts are made to improve the performance in these kinds of platforms. Chemical interaction [14], extended studies of the plasmonics field [15,16] as well as the use of nanotechnologies [17,18] are the groundwork for the development of enhanced devices. Moreover, the detection phase is critical: usually, additional optics or coupling procedures are employed to bring the light signal to off-chip detectors for the electro-optical transduction.

In the path of latest trends, here we present a compact, fully integrated LOC able to detect analyte concentration in a biological sample through an investigation on the optical absorption phenomenon. The goal is to step toward a single chip, completely portable and autonomous: a solution droplet is put in contact with a light-guiding structure and the extent of this interaction is evaluated by monitoring the resulting photocurrent of a monolithically integrated photodetector. More specifically: a glass substrate with SU-8 polymer optical waveguides is used to route light; thin-film hydrogenated amorphous silicon (a-Si:H) photodiodes are monolithically integrated on the substrate to perform on-chip detection, registering a photocurrent related to the optical properties of the solution containing the analyte and, lastly, to the analyte information inside the biological sample. The presented approach intends to simplify the existent systems without sacrificing sensing performance.

## 2. Design and Working Principle

**Figure 1:**
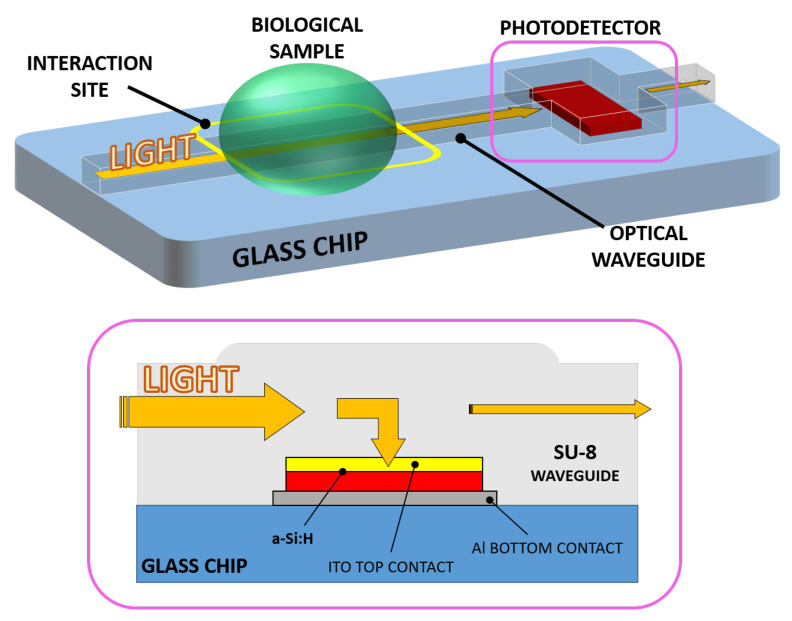
3D sketch of the system (**top**) with a section of the waveguide-detector coupled site (**bottom**).

A novel evanescent waveguide detector has been devised, making use of a SU-8 polymer optical waveguide and an optically coupled amorphous silicon diode as photodetector. The resulting chip consists of a monolithically integrated system-on-glass which performs an interaction with a biological solution and an on-chip detection of the analyte. Light propagating into the device interacts with a biological solution when a sample droplet is put in contact with the waveguide. If the optical properties (in terms of refractive index *n* and extinction coefficient *k*) change with respect to the analyte concentration variation [19,20], the optical power travelling toward the photosensor changes accordingly. As a final step, the electro-optical transduction is carried out by the photodiode. Thus, a change in the analyte concentration corresponds to a shift in the sensor photocurrent. Figure 1 depicts the proposed system and its working principle.

For light propagation, polymer waveguiding structures were considered and, among all, SU-8 was chosen as main material. SU-8 is a versatile, epoxy-based, photosensitive polymer with low electrical conductivity and high optical transparency in the visible spectrum, and is widely used with various micro-electro-mechanical systems (MEMS) [21,22] and microelectronic applications [23,24]. The absorption of light in the ultraviolet (UV) spectrum brings to the possibility of fabricating thin and thick films with high aspect ratios by means of a single UV photolithographic step [25]. Moreover, the SU-8’s high refractive index and low optical losses in a wide frequency spectrum allow the fabrication of waveguiding structures [26,27,28].

The optical detection is performed by a hydrogenated amorphous silicon (a-Si:H) photodiode made of a thin film p-doped/intrinsic/n-doped junction stack. a-Si:H has established itself over the years as leading material for low-cost and large-area applications in the photovoltaic field [29,30] as well as sensing and imaging [31,32]. Among all its features, its low-temperature fabrication process (below 250 ${}^{\circ }$C) makes it very versatile and compatible with the fabrication parameters of metals, polymers and oxides as well as standard complementary metal oxide semiconductor (CMOS) technology. Moreover, its capability of growing in such a low-energy environment, usually by Plasma Enhanced Chemical Vapor Deposition (CVD), makes it very versatile and compatible with a variety of different substrates such as glass, silicon or flexible polymers [33]. Furthermore, amorphous silicon p-i-n junctions are proven to provide high sensitivity in terms of electro-optical responsivity and quantum efficiency across the visible spectrum (from UV to IR), as well as presenting low dark current and, therefore, a low Limit of Detection (LoD) [34]. This a-Si performance, comparable with that of crystalline silicon (c-Si) detectors, together with its versatility, is a key feature in this field to develop smart, sensitive and low-cost biosensing devices [35]. For the proposed system, a (600 × 150) μm${}^{2}$ n-doped/intrinsic/p-doped (nip) stack of a-Si:H was considered to be detection component.

The integration of the two parts was accomplished by considering SU-8 with a double purpose: waveguiding material and, at the same time, electrical insulator around the p-i-n junction, in order to prevent a short circuit to occur between the bottom and top metal layers. The result is an optical waveguide which delivers the travelling light directly to the photodiode by simply overlapping the junction. Moreover, the top electrode of the photodiode must be electrically conductive and optically transparent: indium-tin oxide (ITO) is a transparent conductive oxide (TCO) and is widely used in applications such as photovoltaic and liquid crystal displays (LCD) [36,37]. An ITO thin-film top electrode was chosen with the double task to allow the travelling light to be absorbed by the photodiode while providing electrical connection to the external electronics. The ITO film is finally covered, with a 200 nm-thick titanium-tungsten (Ti/W) alloy film to extract the photocurrent.

In previous investigations [38] the behavior of monochromatic light inside a BK7 ion-diffused waveguiding structure and its coupling into an amorphous silicon p-i-n photodiode through an ITO bottom contact layer was analyzed. Such a system was studied with the goal of developing new solutions in terms of integrated on-chip detection in the biosensing field. In order to find new improvements in terms of versatility, simplicity and fast prototyping, we present a revisited optical guiding part: the double-ion exchange process on the substrate surface was replaced by a single and fast SU-8 photolithographic procedure. Moreover, the original amorphous silicon p-i-n sequenced growth was reversed into a n-i-p stack, in order to collect light from the overlapped SU-8 waveguide through its upper p-doped side.

## 3. Numerical Analysis

**Figure 2:**
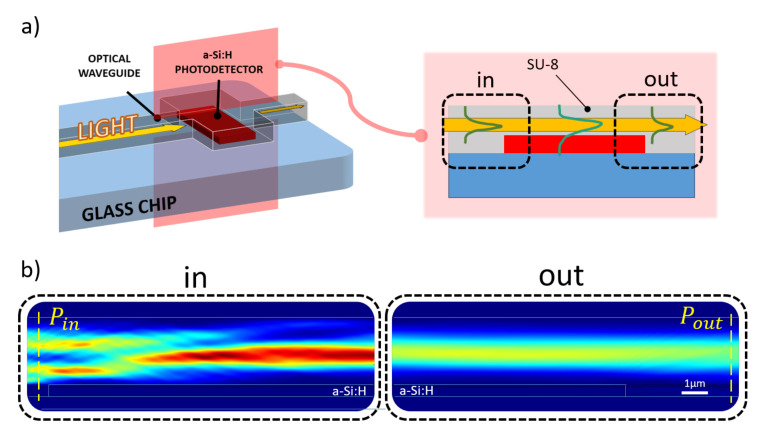
Simulated optical coupling scenario. (**a**) Longitudinal section of the coupling site numerically analyzed to evaluate the amount of optical power absorbed by the diode (a-Si:H) from the overlapped SU-8 waveguide. (**b**) Optical power distribution along the propagation direction (from left to right) light entering (“in” box) and leaving (“out” box) the overlapping site.

A numerical analysis of the proposed system was achieved using COMSOL Multiphysics^®^. The structure described in the previous section was longitudinally sectioned and modeled in a 2-D environment with the “Electromagnetic Waves, Frequency Domain” physics module: the evolution of the electromagnetic field was evaluated along the diode-waveguide overlapped portion with light routed in the SU-8 channel; a 532 nm excitation light was assumed as generic light within the photoemission spectrum of various kinds of biomolecules [39]. The light behavior in such a structure was assessed by means of investigation of optical power flux, represented by the Poynting vector, along the SU-8 waveguide before and after the transit over the diode, as depicted in Figure 2.

The simulation model at the aforementioned frequency, assumed for the glass BK7 substrate a refractive index of 1.52 (as reported by SCHOTT^®^ optical glasses data sheets), while for the 610 nm-thick a-Si:H structure a refractive index of 4.46 and an extinction coefficient of 0.87754 [40] were selected. As top contact, an ITO layer with a refractive index of 2.05 was modeled over the amorphous silicon junction. This value was achieved depositing ITO samples by RF sputtering and measuring their refractive indices with a multi-wavelength ellipsometer. A SU-8 channel with a refractive index equal 1.58 (as reported in MICROCHEM^®^’s “SU-8 3000” data sheet) was considered. The numerical study was achieved varying both ITO and SU-8 thicknesses and determining the optical power absorbed by the amorphous silicon structure with the following equation:

(1) $$CouplingEfficiency\left[\%\right]=\frac{P_{in}-P_{out}}{P_{in}}\cdot 100.$$

**Figure 3:**
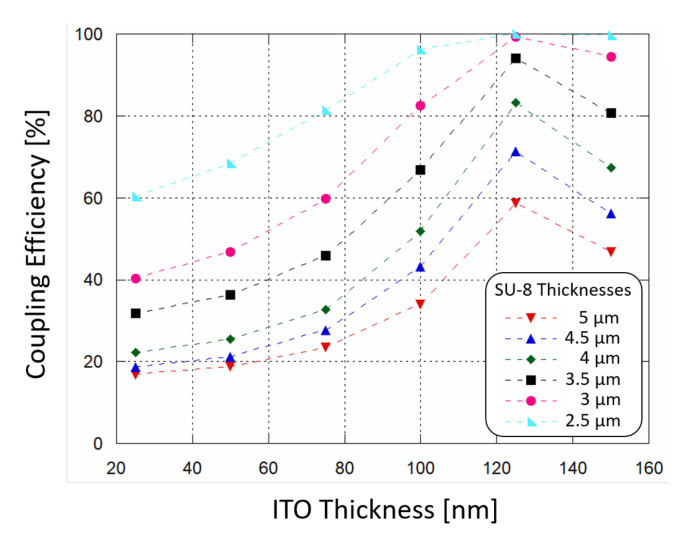
Simulated waveguide-photodiode coupling efficiency as a function of ITO top contact thickness, varying the SU-8 polymer thickness in which light is routed.

The results are plotted in Figure 3: the power absorption (and therefore the coupling efficiency) increases as SU-8 thickness decreases, and the ITO thin film acts as a buffer layer helping the coupling of light. When ITO thickness is around 120 nm the absorption of optical power by the a-Si:H structure reaches a peak. In order to achieve a coupling efficiency higher than 90%, a SU-8 polymer thinner than 3.5 μm and an ITO film thickness around 120 nm must be considered for the fabrication phase.

## 4. Fabrication Process

**Figure 4:**
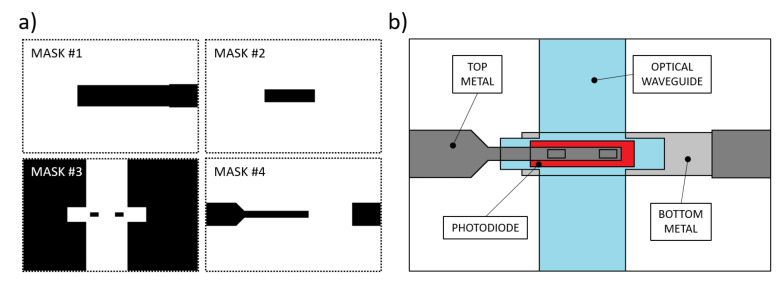
(**a**) Photolithographic masks used for the prototype fabrication. Mask #1: aluminum bottom contact. Mask #2: n-i-p junction patterning. Mask#3: SU-8 insulation layer and optical waveguide. Mask #4: Ti-W top metal. (**b**) Depicted top view of the fabrication result.

The manufacturing procedure for these kinds of devices requires the use of thin film microelectronic technologies, such as Chemical and Physical Vapor Deposition (CVD-PVD), lithographic processes to define the geometries and wet-dry etching techniques to pattern the thin-film deposited layers [41]. Previous studies resulted in a device whose fabrication process required a total of 6 photolithographic masks: in addition to the waveguides masks, glass-etched markers were made to align the optics with the sensor [38]. Here we present a simplified process flow, consisting of a total of 4 lithographic steps, with the masks depicted in Figure 4a. Figure 4b depicts a qualitative top view of the fabricated device.

**Figure 5:**
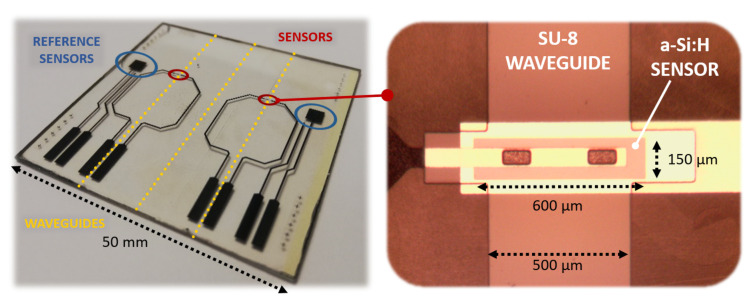
Fabricated prototype (**left** side) with an enlargement on the photodiode with the overlapped SU-8 waveguide (**right** side).

A 200 nm-thick Aluminum film was deposited on a glass substrate by thermal evaporation in a Balzers 510 evaporation system, and patterned according to mask #1. A three-chamber ultrahigh vacuum system hosted the substrate to grow a phosphorus-doped/ intrinsic/boron-doped (n-i-p) a-Si:H stack by Plasma Enhanced Chemical Vapor Deposition (PECVD) with thicknesses equal to 100 nm/500 nm/10 nm, respectively; subsequently, RF Sputtering was selected to deposit a 100 nm-thick ITO film over the amorphous silicon stack. Mask #2 was used to pattern the junction body with its top ITO contact. A 5 μm-thick SU-8 layer was spin-coated and UV-exposed according to mask #3 to define the optical channel and the insulation layer around the junction, with two via-holes over the ITO top contact. A 250 nm-thick Ti-W layer was deposited by RF sputtering to obtain an electrical connection through the via-holes to the pads at the substrate edge. Mask #4 was used to define its geometry. Figure 5 shows a (50 × 50 ) mm${}^{2}$ glass substrate hosting two (600 × 150) μm${}^{2}$ a-Si:H sensors fabricated with the corresponding overlapped 500 μm-wide SU-8 optical channels. Moreover, two (3 × 3) mm${}^{2}$ a-Si:H photodiodes were placed as reference sensors to act as a support for an electro-optical characterization of the system.

## 5. Electro-Optical Characterization

Together with the evanescent waveguide detecting diodes (Figure 5, right enlargement), two (3 × 3) mm${}^{2}$ testing n-i-p structures, showed in Figure 5 as “reference sensors”, were fabricated on the same substrate and during the same deposition runs. The testing junctions, which do not have an overlapped waveguide and are placed at a 5 mm distance, were also employed to perform an optoelectronic characterization of the system.

### 5.1. Photodiode: Electro-Optical Characterization

**Figure 6:**
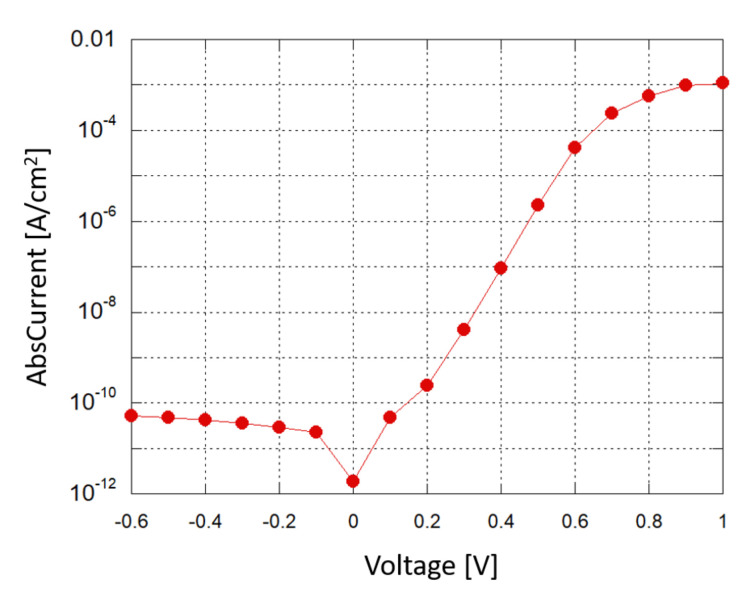
Photodiode: current density vs. applied voltage in a semi-logarithmic plot.

A Source-Measuring Unit (SMU) was used to obtain a current–voltage (I–V) characterization of the photodiode in dark conditions; Figure 6 shows the results in a semi-logarithmic plot. When a small reverse bias voltage is applied (−0.1 V), the reverse saturation current density is around 2 ×10${}^{-11}$ A/cm${}^{2}$, corresponding to a shot noise current contribution of about 2.5 fA/$\sqrt{\mathrm{Hz}}$, which is below the minimum detectable signal (MDS) in our experimental setup.

The photodiode response at the simulated wavelength was assessed by sending a 532 nm laser beam orthogonally with respect to the n-i-p-structures. The test was carried out measuring the beam’s optical power, using a power meter, with an iris diaphragm to vary the light reaching the specimen by setting different beam widths. A translation stage was used to align the beam to the diodes, and the reference diodes as well as the sensing diodes were involved in the characterization. The diodes’ photocurrents were measured with the SMU. As a result, the sensors’ electro-optical Responsivity (R) was evaluated as a ratio between the generated photocurrent and the impinging optical power at the considered surfaces: photocurrents of about 4.95 μA were registered for a 10 μW impinging light beam, leading to a R value of 495 mA/W.

### 5.2. SU-8 Waveguide: Optical Characterization

**Figure 7:**
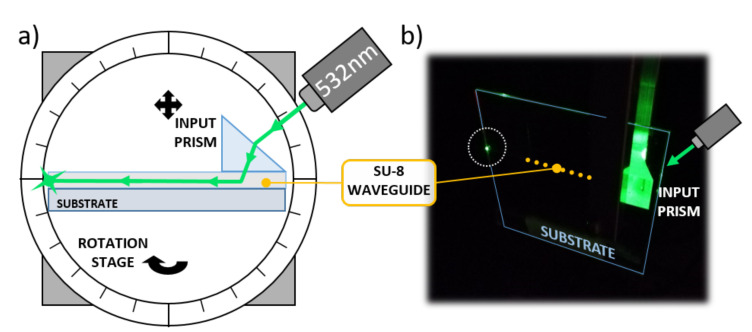
Optical waveguide characterization. (**a**) Characterization setup. (**b**) Picture taken during the optical tests.

A glass substrate with 500 μm-wide optical waveguides was tested to investigate the SU-8 guiding performance. An input high refractive index SF6 prism (n=1.8157 at 532 nm) was used to couple light into the optical channel, as depicted in Figure 7.

The substrate was mounted on a rotation stage to finely adjust the impinging angle of the laser beam. A coherent Fieldmaster GS Laser Power and Energy Meter was used to register the optical power entering and exiting the SU-8 channel. As a result, the propagation loss presented a value of about 0.6 dB/cm, comparable with similar polymer waveguides found in the literature [42,43].

### 5.3. Waveguide-Photodiode Optical Coupling Test

**Figure 8:**
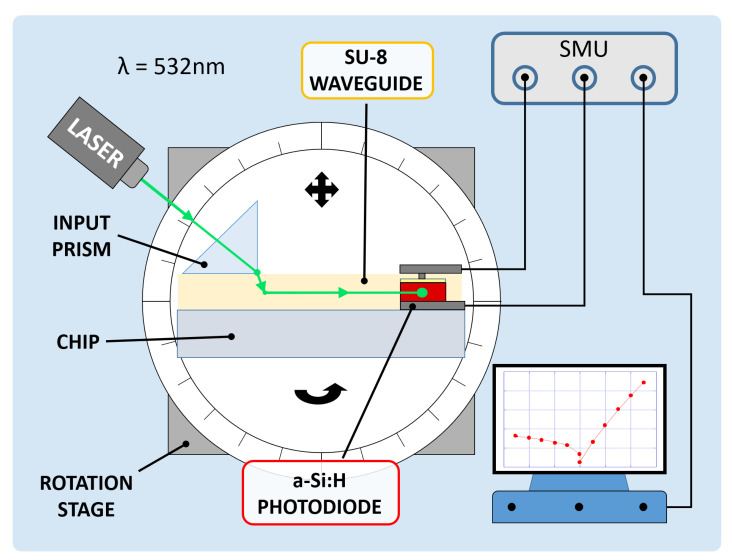
Electro-optical characterization setup.

**Figure 9:**
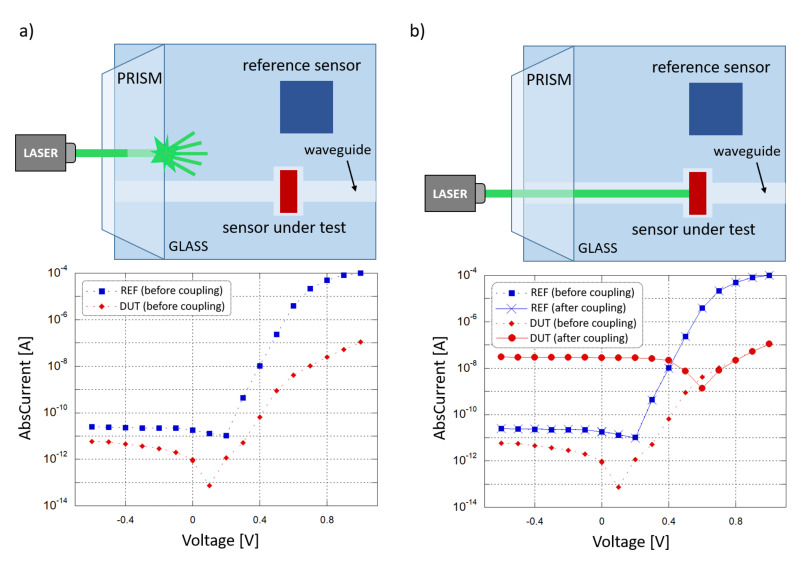
Optical coupling test. (**a**) Laser beam not aligned to the DUT waveguide (scenario depicted at the top): current-voltage behavior of the two sensors (plotted at the bottom). (**b**) Laser beam aligned to the DUT waveguide (scenario depicted at the top) current-voltage behavior of the two sensors (plotted at the bottom).

A coupling test was performed to observe the sensor capability to collect light propagating in the corresponding waveguide. For this purpose, a high-index optical prism was employed to couple a 532 nm laser light source into the system. The entire glass chip was then mounted onto a rotation stage to adjust the angle of incidence of the laser beam and optimize the optical coupling. Figure 8 depicts the characterization setup. A (600 × 150) μm${}^{2}$ diode with an overlapped 500 μm-wide SU-8 waveguide was considered to be diode under test (DUT); a (3 × 3) mm${}^{2}$ diode, without an overlapped waveguide and placed at a 5 mm distance, was considered to be reference diode (REF). The test was performed to study the optical coupling between the DUT and the corresponding waveguide. The REF monitored the noise contributions due to scattered and diffused light phenomena. The two photocurrents were measured by a Keithley 236 SMU and monitored in 2 conditions. First, the laser beam and the prism were coupled with an optimized angle, but the light beam was not aligned to the waveguide and hit the glass substrate between the DUT and the REF, as depicted in Figure 9a. Subsequently, the laser beam and the prism were favorably coupled, and the light beam was aligned to the DUT waveguide, as depicted in Figure 9b.

Figure 9a shows the I-V curves where a shift is present in the DUT and REF open-circuit voltage ($V_{OC}$) with respect to the dark condition: this is caused by photocurrent generated by the light scattered from the prism into the substrate, and was considered to be background noise. As shown in the two plots, when the condition changes from “uncoupled” to “coupled” the noise contribution remains almost the same, as monitored by the REF, but a significant variation in the DUT photocurrent has been observed, from 900 fA to 28 nA at 0 V bias, showing an increment of about 4 orders of magnitude. Considering also that the REF is far more sensitive to scattered light, with a 100 times bigger active area than the DUT, these results confirm the successful on-chip light coupling from a SU-8 waveguide to our thin-film a-Si:H photosensor.

## 6. Proof of Concept: Hemoglobin Concentration Detection

Hemoglobin (Hb) solutions were analyzed as a simulated scenario for a sensing demonstration in the blood analysis field. Hb, as a protein in red blood cells responsible for oxygen transportation, is a marker for the monitoring of many physiological functions in the human body. Hb values can change due to human body adaptation to particular environments [44]. Under a certain concentration in blood, anemia is diagnosed [45,46]. It can be used as marker to study the longevity of older people [47,48] and chronic diseases [49]. Pregnancy issues such as maternal and perinatal mortality, as well as preterm birth and low birth weight can be caused by Hb imbalances or deficiencies in pregnant women’s blood [50]. Moreover, Hb can be an indicator of inadequate diet and certain food deficiencies [51]. Higher and lower Hb concentrations with respect to optimal values have been correlated with cardiovascular diseases and coronary heart diseases [52]. Blood is also an increasingly studied subject in sports [53]: the evaluation of athletes’ blood profiles such as red cells volume, plasma content, blood cell concentrations and oxygen transport indicates Hb as key in examining physical exercise, dehydration phenomena [54], cardiovascular and respiratory function in aerobic performance [55,56]. Full blood exams in sports were introduced with the aim to protect athlete health and, at the same time, to detect blood adulteration and doping procedures [57]. Hb can be manipulated for maximal exercise capacity: the result is an increase in the red cells mass and an enhancement in oxygen transport capacity, producing an improvement in athletic response in terms of stamina, endurance and overall performance [58,59].

Blood has been extensively studied over the years at various frequency ranges: Friebel et al. [19,60] characterized the optical properties of Hb solutions at different concentrations in the visible spectrum. These data, at a 532 nm wavelength, were extracted and used to model Hb-water solutions in a simulated sensing demonstration, carried out with COMSOL Multiphysics^®^. In this scenario, a longitudinal section of the biological interaction site, consisting of a SU-8 channel over the glass substrate and overlapped by the biological solution, was modeled. To evaluate the evolution of the electromagnetic field, the “Electromagnetic Waves, Frequency Domain” physics module was employed. As initial excitation in the optical waveguide, 532 nm light was assumed. The propagating light was monitored, in terms of optical power flux, along the SU-8 channel before and after the transit into its overlapped portion with the Hb droplet. The extracted refractive index *n* and extinction coefficient *k* of 5 solutions were implemented, corresponding to 4.6%, 10.4%, 16.5%, 28.7% and 32% Hb percentage. In addition to this, plain water, corresponding to a 0% concentrated solution, was assumed as sixth sample. Table 1 resumes the implemented values.

**Figure 10:**
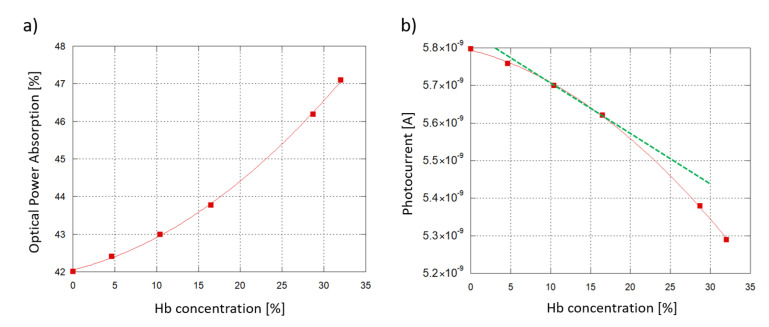
Sensing demonstration: 6 hemoglobin-water samples at different concentrations interacting with the SU-8 waveguide. A 6 mm-long interaction site and a 5 μm-thick SU-8 waveguide are considered. (**a**) Modeled optical power absorption from the sample plotted as a function of the Hb concentration in water. (**b**) a-Si:H diode photoresponse vs. Hb concentration.

Figure 10a plots the optical power absorption by the 6 samples as a function of the Hb concentration in water when an 6 mm-long interaction site (see Figure 1) and a 5 μm-thick SU-8 waveguide are considered. The optical absorption is defined as the difference between the optical power of the travelling light evaluated before and after its transit under the interaction site. As the Hb concentration increases, the sample absorbs more of the underlying travelling light, with a quadratic trend.

To investigate the sensor response, an interpolation was carried out between the simulated resulting optical power values and the experimental Responsivity R measured at 532 nm. An initial working photocurrent of 10 nA was assumed as initial reference state, without any biological solution interacting with the waveguide. Taking into account a 60% coupling efficiency (when the ITO thickness is around 120 nm, see Figure 3) and the value of R, the correspinding optical power routed towards the diode was about 33.3 nW. Figure 10b plots the expected photocurrent, showing a similar, quadratic trend. As an additional insight, Hb concentrations from 10.4% to 16.5% were considered to be values within the typical range of interest for blood analysis. Inside this range, a linear approximation has been considered (Figure 10b, depicted in green) with the following trend:

(2) $$I_{ph}=5.8339^{-9}-1.2852^{-11}\cdot c_{Hb}$$

where $I_{ph}$ is the diode photocurrent when the Hb solutions are put in the interaction site, and $c_{Hb}$ is the modeled Hb concentration in water.

Inside the analyzed range, the sensor photocurrent varies from 5.70 nA (10.4% Hb) to 5.62 nA (16.5% Hb), with a span ΔI equal to 78.2 pA corresponding to a Hb concentration span Δc of 0.061. From these values we derive that the device Sensitivity (S), obtained as ΔI/Δc, is equal to 18.2 pA/(g/dL). To correctly determine the LoD, noise must be taken into account. In fact, the discreteness of the electron charge causes time-dependent fluctuations in the junction-based components’ electrical current [61], with a mean square value equal to 2qIB, where *q* is the elementary electrical charge and *B* the signal band. The Schottky noise current contribution of our system, considering that our current measurements were taken with a frequency band of 1 Hz, is around 42.7 fA. As a consequence, a Minimum Detecting Signal (MDS) equal to three times the noise, 128 fA, was assumed. The LoD was then calculated as:

(3) $$LoD=MDS\cdot \frac{\Delta c}{\Delta I}$$

As a result, the proposed system’s theoretical LoD is approximately 100 ppm in the considered range.

**Figure 11:**
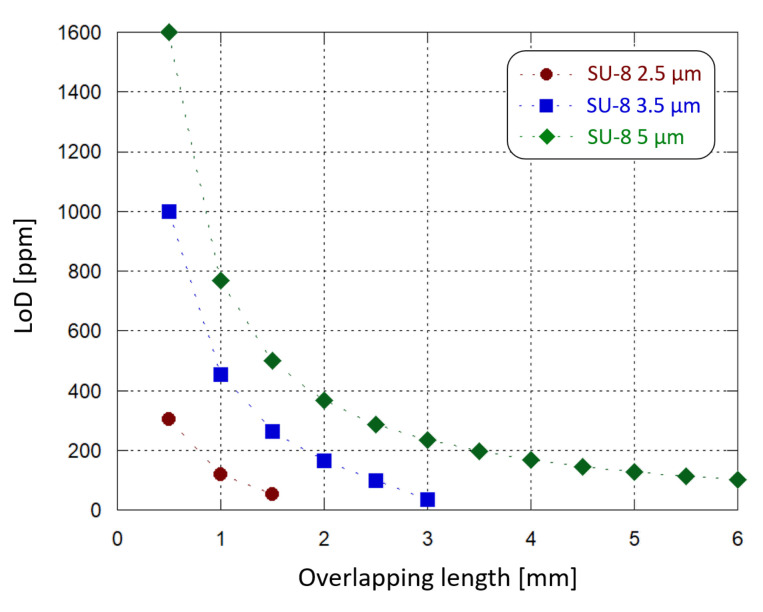
Sensing demonstration: limit of Hb concentration detection (inside the considered 10.4–16.5% range) by the developed system as a function of the waveguide-sample overlapping length, for different SU-8 waveguide thicknesses.

Similar simulations were carried out varying the thickness of the SU-8 waveguide and the length of the interaction site. We found that the optical power absorption increases by decreasing the SU-8 thickness and increasing the droplet diameter. In particular, for each Hb concentration it is possible to design a combination of SU-8 thickness and interaction site length where 100% of the light is absorbed. In this condition no optical power reaches the detector and the photogenerated current is equal to zero. For example, we have found that considering a 2.5 μm-thick SU-8 waveguide, an interaction site longer than 2 mm gives 100% of light absorption. Instead, if a 1 mm-long interaction area is modeled, both 2.5 μm- and 5 μm-thick SU-8 waveguides can be implemented without losing 100% of the optical power: different thicknesses correspond to different absorption ranges, but with a similar quadratic trend. Figure 11 plots the limit of Hb concentration detection achieved by the sensor, with 10 nA of initial operating current, as a function of the sample-waveguide overlap length, for 3 different SU-8 thicknesses. The overlap length is varied from 0.5 mm to 6 mm with 0.5 mm steps. When a 2.5 μm-thick SU-8 is chosen, a 2 mm-long sample-waveguide overlap causes the whole propagating light to be absorbed by the solution. The same phenomenon occurs with a 3.5 μm-thick SU-8 waveguide when the interaction site is 3.5 mm-long. LoD values around 100 ppm can be achieved in multiple ways: by selecting a 2.5 μm-thick SU-8 and a 1.5 mm-long interaction site, or a 3.5 μm-thick SU-8 and a 3 mm-long interaction site, or a 5 μm-thick SU-8 and a 6 mm-long interaction site.

A thinner waveguide provides more sensitivity in the interaction with the sample under test; at the same time, the waveguide-solution overlap surface must also be taken into consideration as a key factor to choose an optimized absorption range, which leads to optimized sensing performance. In this case the waveguide width was fixed, and the length was tuned. Moreover, they must be adjusted to not dissipate into the solution the whole propagating light and, therefore, to avoid sensing anomalies. Furthermore, a LoD below 100 ppm could be achieved by finely tuning also other parameters (such as the waveguide width and the photojunction’s stack thickness), taking also into account practical considerations: for example, a thinner SU-8 layer could improve the waveguide sensitivity but providing a worse electrical insulation to the photodiode.

As an additional note, both interaction length and waveguide thickness can be finely tuned to adapt the system to different scenarios and to host diverse kinds of biological solutions, depending to their optical properties, making it a versatile optical biosensing platform.

## 7. Conclusions

This paper provides a study on a compact, inexpensive and easy-to-use Lab-on-Chip, where the concentration of an analyte in a biological sample is evaluated through the interaction of a solution droplet with a light-guiding structure and the detection of the optical absorption with an on-chip a-Si:H photodiode. The endeavor towards a “true” LOC is accomplished by the integration of the detection element into the chip where the biological interaction takes place. A simulation campaign was arranged to study the coupling of light between the amorphous silicon structure and the overlapped SU-8 waveguide. The results were taken into consideration for the fabrication phase to maximize the optical coupling efficiency. The optical characterization of the waveguide was performed together with a voltage-current electrical characterization of the n-i-p photodiodes. A coupling test examined the system behavior with 532 nm excitation light. As a result, photocurrent increases by 4 orders of magnitude when light is coupled into the optical waveguide.

As proof of concept, hemoglobin was used as the target analyte for sensing and simulation tests. Different parameters, such as the waveguide thickness and the interaction site length, can be tuned to adapt to the analysis requirements and to optimize the sensor sensitivity. Preliminary results indicate that for the studied application, the limit of detection can reach 100 ppm, in line with colorimetric methods currently on the market, encouraging further developments in implementing these kinds of systems in the biomedical field.

## Figures and Tables

**Table 1:** Refractive Indices *n* and Extinction Coefficients *k* of Hb solutions in water [19,60].

Hb Concentration in H${}_{2}$O [%]	*n*	*k*
0	1.334	1.32 × 10${}^{-9}$
4.6	1.346	2.36 × 10${}^{-4}$
10.4	1.361	6.13 × 10${}^{-4}$
16.5	1.379	1.00 × 10${}^{-3}$
28.7	1.409	1.80 × 10${}^{-3}$
32.0	1.419	2.00 × 10${}^{-3}$

## Data Availability

Not applicable.
